# *Cryptosporidium* and Cryptosporidiosis: The Perspective from the Gulf Countries

**DOI:** 10.3390/ijerph17186824

**Published:** 2020-09-18

**Authors:** Shahira A. Ahmed, Panagiotis Karanis

**Affiliations:** 1 Department of Parasitology, Faculty of Medicine, Suez Canal University, Ismailia 41522, Egypt; Shahira_ahmed@med.suez.edu.eg; 2 Medical Faculty and University Hospital, University of Cologne, 50937 Cologne, Germany; 3 Department of Basic and Clinical Sciences, University of Nicosia Medical School, CY-1700 Nicosia 24005, Cyprus

**Keywords:** *Cryptosporidium*, prevalence, incidence, human, Arabic Gulf, animal, water, food, air

## Abstract

The present review discusses the burden of cryptosporidiosis in the Gulf Cooperation Council (GCC), which is underreported and underestimated. It emphasizes that the *Cryptosporidium* parasite is infecting inhabitants and expatriates in the Gulf countries. Children under 5 years are a vulnerable group that is particularly affected by this parasitic disease and can act as carriers, who contribute to the epidemiology of the disease most probably via recreational swimming pools. Various risk factors for cryptosporidiosis in the GCC countries are present, including expatriates, predisposing populations to the infection. Water contamination, imported food, animal contact, and air transmission are also discussed in detail, to address their significant role as a source of infection and, thus, their impact on disease epidemiology in the Gulf countries’ populations.

## 1. Introduction

Cryptosporidiosis is a significant diarrhoeal disease for both people and animals worldwide. Several species of the protozoan parasite *Cryptosporidium* can cause this disease [1], in which *Cryptosporidium* oocysts have ubiquitous presence in the environment. *Cryptosporidium* oocysts transmission can occur following direct or indirect contact with an infected host usually via the faecal–oral route. Person-to-person contact, zoonosis, and the consumption of contaminated food or water are well known mechanisms for faecal–oral transmission [2,3], with a significant risk of infection from the ingestion of a single oocyst [4]. When the oocysts enter the gastrointestinal tract, the invasive *Cryptosporidium* causes damage to the small intestinal epithelium. It disrupts the barrier function and absorption capability that leads to mild-to-severe diarrhoea and other abdominal symptoms. In immunocompetent adults, *Cryptosporidium* infection is usually asymptomatic or mild, which is generally self-limiting.

Currently, *Cryptosporidium* has 41 reported species with more than 60 valid genotypes [5]. Amongst them, 21 species and genotypes have been identified in humans, out of which *C. parvum* and *C. hominis* are the most common pathogenic species, causing more than 90% of infections in humans. *C. meleagridis*, *C. ubiquitum*, *C. cuniculus*, *C. muris*, and *C. andersoni* are other pathogenic species that have sporadically emerged in human cases of zoonotic outbreaks, especially when there has been direct contact with infected animals [6,7,8,9,10].

The disease-causing species and their associated subtypes have contributed to a substantial global burden of cryptosporidiosis and play a role in the severity of the disease [11]. Globally, diarrhoeal diseases have killed 1.6 million people in 2017. One third of these deaths were children under 5 years, and their highest mortality are from sub-Saharan Africa (SSA) and South Asia. This was because of unsafe drinking water and poor sanitation [12]. *Cryptosporidium* oocysts can transmit through water, making it one of the most important causes of human and livestock infectious diarrhoea [13,14,15]. Contamination with *Cryptosporidium* has been identified as the leading cause of 905 worldwide waterborne outbreaks [13,16]. Additionally, it has been reported to be responsible for more than 8 million cases/year of foodborne illness and 25 documented foodborne outbreaks [17,18]. After 2000, various Latin American countries have reported that *Cryptosporidium* spp. were the most prevalent parasites in their water samples [14]. Oocyst contamination has also been found in various water sources from African countries, with a high incidence in half of the reports (60/120) [19]. Poly-factors have been reported in the literature that highlight the predisposition for the distribution of waterborne protozoa (e.g., *Cryptosporidium*) in these countries. Weak institutional infrastructure, political conflict, inadequate water supplies, unclean water, underdevelopment, poverty and illiteracy, population density, high levels of malnutrition, social unrest, poor hygiene and sanitation, climate change, and water crises have all been cited as negative factors that are promoting cryptosporidiosis and other diarrhoeal diseases [19,20,21].

Developing countries (low resources category) are particularly more affected from diarrhoeal diseases caused by *Cryptosporidium* infection [16,19,20,22,23,24]. The Global Enteric Multicentre Study identified *Cryptosporidium* at seven sites in SSA and South Asia as one of the four major contributors to moderate-to-severe diarrhoeal diseases during the first 2 years of life at all sites [25]. From the African perspective, *Cryptosporidium* has been considered as one of the leading causes of diarrhoea in childhood with dramatic adverse effects on their growth and development [20]. In the Asian countries, a plethora of reports has addressed the occurrence of *Cryptosporidium* infection from different species, where West Asia (among them SA, Kuwait, and Oman) constituted 33% of these reports [22]. Regarding mortality caused by *Cryptosporidium* in SSA and South Asia, it has been estimated to contribute to approximately 202,000 deaths per year. [26]. In Kenya, Mali, Mozambique, Gambia, and South Asia, *Cryptosporidium* infection has caused a higher risk of death in toddlers aged 1–2 years with moderate-to-severe diarrhoea (4%) [25]. The critical absence of governmental surveillance systems to document parasitic protozoan diseases, particularly *Cryptosporidium* in humans, the environment, and food and water sources, has led to significant underestimation of reported waterborne diseases and associated outbreaks in those developing countries [19].

In developed countries (high resources category), *Cryptosporidium* infection has risen to become a serious problem. In the Netherlands, a country with high hygienic standards, the burden of *Cryptosporidium* according to the calculated Disability-Adjusted Life Years (DALYs) has increased from previously estimated reports. *Cryptosporidium* disease burden has produced 137 DALYs and a cost of 19.2 M€ from illness in 2017, whereas long-term manifestations, including recurring diarrhoea and joint pain, accounted for 9% of the total DALYs in the Netherlands [27]. In the United States of America (USA), which constitutes the world’s largest economy, a high number of *Cryptosporidium* cases have been reported. According to the Center for Diseases Control and Prevention (CDC), the US had a fourfold increase in the number of *Cryptosporidium* outbreaks from 2014 to 2016 [28]. Contaminated water supplies and/or food sources are mostly the source of *Cryptosporidium* infection in developed countries [29,30]. There is therefore an urgent need to prioritize *Cryptosporidium* contamination with regard to public health surveillance. The operational surveillance system in developed countries mainly focuses on infections that are caused by bacteria and viruses and only include a limited number of parasitic protozoa in their surveillance programs. Frequently, these protozoa are neglected as the main cause of diarrhoea symptoms in high-income countries due to an overconfidence in public hygiene, municipal sanitization services, and good agriculture and livestock practice [31].

The Gulf Cooperation Council (GCC) is a political and economic alliance between six Middle Eastern countries, namely, Bahrain, Kuwait, Oman, Qatar, Saudi Arabia (SA), and the United Arab Emirates (UAE) [32]. The GCC countries (intermediate resources category) are concurrently classified as high income and as developing countries [33]. The GCC countries face numerous environmental challenges (water scarcity, water quality, desertification, and air and marine pollution) that require reconciliation of many conflicting priorities [34,35].

One of the most critical problems that affects public health in the GCC countries is the lack of renewable water resources. Infrequent rainfall in the Arabian Peninsula has led to the overutilization of ground water resources that has consequently affected the qualitative and quantitative of ground water needed for agriculture, industry, and personal consumption [36]. Most of the demand for fresh water in the GCC countries relies on desalination of seawater, which is a process that requires an extensive pre-treatment and conditioning of seawater [37]. Due to this rigorous treatment of seawater, researchers typically do not suspect desalinated drinking water as a source of *Cryptosporidium* contamination; however, it can happen. During the course of the water treatment process, contaminants and beneficial nutrients could be removed and of course some might be added [38] once stored in tanks or used to fill swimming pools [39,40].

The burden of infectious diarrhoea in the countries of the GCC has been addressed in various reviews on the Middle East and North Africa (MENA) and Eastern Mediterranean Region (EMR). Infectious diarrhoea has been reported by the United States military after it experienced a significant burden from this disease in the MENA campaigns of World War II [41,42]. Traveller’s diarrhoea due to ingestion of bacteria, viruses, or protozoa has been reported to affect travellers to Saudi Arabia [43]. Diarrhoeal infections among MENA children pose a significant public health challenge [44] and has been indicated in many reports affecting children in the GCC countries [39,45,46,47,48,49,50,51].

The mortality from diarrhoeal diseases in the GCC countries has also been estimated in a study from the EMR. It has been estimated that over 103,692 deaths have occurred in 2015 due to diarrhoea in the EMR. The majority of these deaths (63.3%) have occurred in children under 5 years and the DALYs/100,000 ranged from 304 in Kuwait to 38,900 in Somalia [21]. *Cryptosporidium* has been reported to be the 7th leading cause among 13 diarrhoeal aetiologies responsible for death in the EMR population. Approximately 4569.06 death have resulted in children under 5 years and 4796.2 death from all age groups due to fatal cryptosporidiosis in the EMR area. It has been noted that mainly UAE and Kuwait have the lowest prevalence-weighted risk for diarrhoeal infection [21].

The wealth of the GCC countries has attracted many people to seek work opportunities that has notably increased the population in the region and subsequently increased the burden of infectious diseases, particularly gastrointestinal diseases [47,52]. The pattern of the parasitic infection has shifted to reflect this newly mixed population (inhabitants and immigrants), whereas many of these immigrant nationals have dissimilar educational backgrounds, varied eating habits, different religious beliefs and cultural practices [47].

Little is known about the true extent of intestinal parasitic infection, particularly cryptosporidiosis, among the inhabitants of the GCC countries. Several studies have reported intestinal parasites infection in immigrant food handlers, labourers, and hospitalized children from this region [39,45,46,47,48,49,50,51]. Economic migrants seeking employment in the GCC countries (e.g., servants, food handlers, housekeepers, childcare assistant, and labourers) may arrive carrying their parasitic infections with them. Therefore, the risk of parasitic infection has been estimated to be higher in some sectors of the communities, especially asymptomatic carriers who are employed in the food industry [53].

The food industry has been suspected to be the greatest threat in the spread of diarrhoeal aetiologies. The GCC countries import large amounts of food in order to bridge the gap between food production and food consumption. Imported food mainly comes from high-risk countries with a known epidemiology of diarrhoeal diseases [35]. Imported leafy greens and other fresh produce are highly suspected to be a vehicle for the transmission of the *Cryptosporidium* infection [18,54,55]. The potential for food contamination on and off farms is high since it could be produced or washed with contaminated water. Infected food handlers are another common source of *Cryptosporidium* contamination in the food chain imported by the GCC countries [17,18].

Studies from countries with low, intermediate, and high resources have identified *Cryptosporidium* as one of the major causes of diarrhoea and childhood malnutrition [56]. The magnitude and nature of environmental threats might be the link with the incidence of cryptosporidiosis burden and might explain the differences between the previous three categories.

In this context, immigrant geographic origin, globalization of food supply to meet the demand of the increased labour force, food and water contamination, climate change, as well as poor hygiene after direct animal contact have all contributed to the annual flux in *Cryptosporidium* transmission and infection rates within the GCC countries [54,57].

In the present review, we aim to discuss the size of the burden of *Cryptosporidium* infection in the GCC countries based on the existing information, and to discuss the risk factors that contribute to the *Cryptosporidium* infection in such a wealthy region.

## 2. Methods

### 2.1. Search Strategy

The PubMed, Science Direct, and Scopus databases were searched with no restriction to language or year of publication. To evaluate the burden of *Cryptosporidium* in the GCC population, a clear description of the questions raised with regard to participants, interventions, conditions, outcomes, and study design (PICOS) was performed. The literature search strategy was limited to title/abstract/keyword using the following MeSH terms/key words: (*Cryptosporidium* OR Cryptosporidiosis OR Parasite) AND (Infection OR Prevalence OR Incidence OR Occurrence OR Burden) AND (Human OR Animal OR Water OR Food) AND (Bahrain OR Kuwait OR Qatar OR Saudi Arabia OR Oman OR United Arab Emirates). The screened articles were published between 1971 and 2020. Some relevant articles that were published in Arabic local journals have been retrieved from Library Genesis Scientific Articles and Egyptian Knowledge Bank, Google Scholar, Iraqi Scientific Academic Journals, and ResearchGate.

### 2.2. Inclusion and Exclusion Criteria

Retrieved articles with titles that suggested the topic of *Cryptosporidium* in humans were screened and selected as part of the eligibility for inclusion in the literature review. Abstracts from the selected reference titles were reviewed to determine if the selected studies have met the inclusion criteria. Review of an entire article was performed based on the selected abstracts that previously met the inclusion criteria. The exclusion criteria consisted of studies on animal cryptosporidiosis or studies that related to foodborne/waterborne cryptosporidiosis as they will later be detailed in the risk factors chapter. The articles that have been published in English or Arabic were the only selected languages included in the review. Articles in the form of case reports or reviews or conference proceedings were excluded.

For each article, the following information was extracted: location of the study, type of residents, *Cryptosporidium* detection method, participants classification, most affected age of participants, symptoms associated with the disease (when available), number of cases, and prevalence of the disease—as reported by the authors or calculated from data presented in the paper (when available).

## 3. Results and Discussion

**Figure 1:**
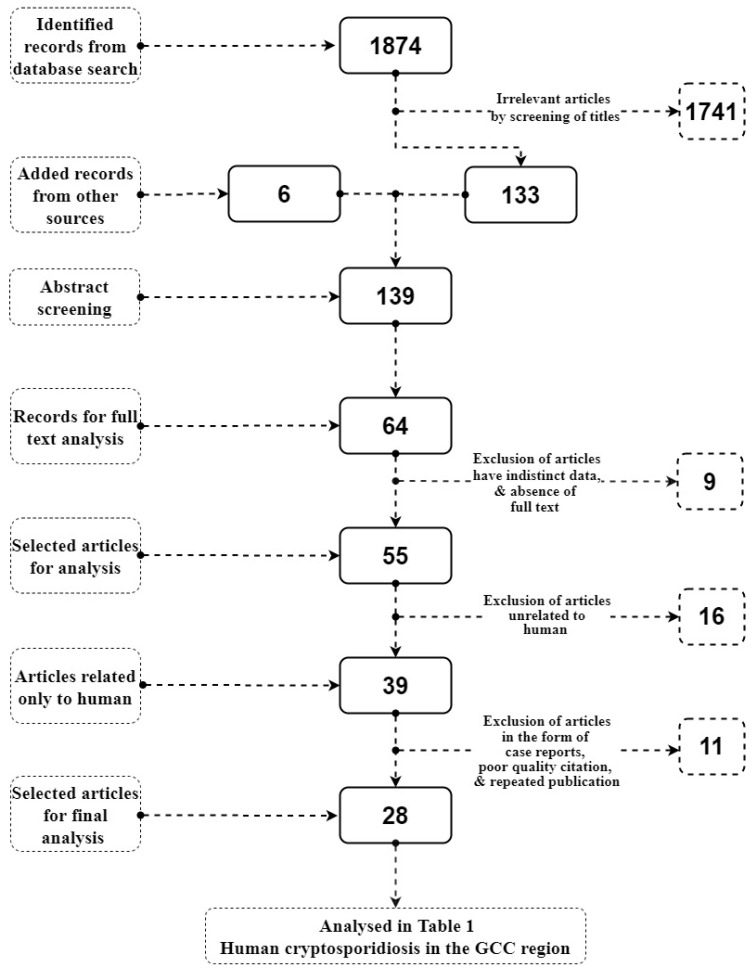
Results profile of the selected articles.

The combined search retrieved 1874 studies. A total of 133 studies were retained based on screening of the titles. An additional six studies were added by the screening reference lists from other sources. Therefore, 139 studies were subjected to abstract screening. In total, 64 articles were retained for full text analysis and subsequently 39 articles were selected for the analysis of human cryptosporidiosis, from which only 28 of the articles were selected for final analytical inclusion (Figure 1). Due to incompatibility with the inclusion criteria, 36 articles were excluded. Specifically, the exclusion criteria were based on articles that had indistinct data, absence of full text, poor quality citation, reviews, case reports, or reports that included the same results as another paper published by the same author.

**Figure 2:**
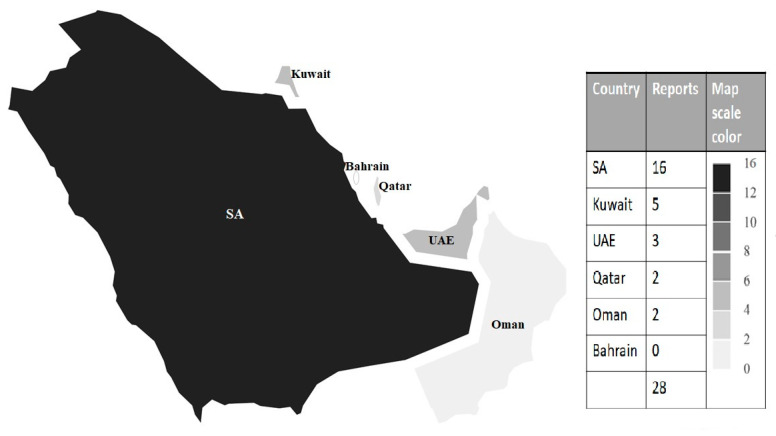
Distribution of *Cryptosporidium* reports in the GCC countries.

Out of the six GCC countries, five countries have reported human infection from *Cryptosporidium* spp. Saudi Arabia leads other GCC countries in the reporting of *Cryptosporidium* infection. Bahrain has not issued any reports concerning *Cryptosporidium* infection (Table 1, Figure 2).

The allocation of *Cryptosporidium* reports in the GCC countries is presented in Figure 2. The burden of *Cryptosporidium* in the GCC countries is presented in Table 1. Molecular genotyping and sub-typing data of *Cryptosporidium* in Gulf reports are presented in Table 2. The situation of *Cryptosporidium* in water resources of the GCC countries is summarized in Table 3. Information on the *Cryptosporidium* occurrence in animals within the GCC countries is tabulated in Table 4. The results that are indicated in the figure and tables are described below.

### Burden of Cryptosporidium in the GCC Countries

The six GCC countries are classified as high-income developing countries that share an infection prevention and control program [58]. Other public health programs have been declared successful by the World Health Organization (WHO) [47].

Only 28 reports of cryptosporidiosis have been published from 5 of the 6 GCC countries. Considering that many of these wealthy countries have the necessary research equipment and facilities, the number of reported articles is considerably low for their capability. This situation indicates an underestimation and underreporting of *Cryptosporidium* infection in the Gulf region.

Saudi Arabia has the highest number of reported *Cryptosporidium* infections in humans with a significant *p*-value < 0.05 in comparison to the rest of the GCC countries. Saudi Arabian reports of *Cryptosporidium* infection have formed half of the total reports number (16/28) cited in the literature that reached an incidental rate of 50% (Figure 2, Table 1). The Kingdom of SA is considered to be the largest of the GCC countries with a population of 28.5 million people [33].

It has a well-established public health system and public safety measures that are applied before mass gatherings that attempt to protect pilgrims during the Hajj season. Gastrointestinal infections during mass gatherings are a major health hazard. Therefore, SA authorities routinely provide continuous surveillance for several protozoal, viral, and bacterial pathogens as a part of its measures to protect public health [59]. The proactive safety measures and awareness of infectious disease has placed Saudi Arabian authorities higher in the reporting scheme within the GCC countries.

Even though the Saudi government has key planning considerations for emerging diseases alerts based on the WHO’s recommendations, *Cryptosporidium* infections has been reported within the population of Makkah before and during the Umrah season. It has been observed that the incidental rate of various intestinal parasites has increased by 7.5% among people around the Holy Masjid during Umrah [60]. Overcrowding has been frequently cited as a significant risk factor associated with *Cryptosporidium* infection in other low- and middle-income countries [29].

The number of *Cryptosporidium* reports from the other GCC countries (Kuwait, UAE, Qatar, and Oman) varied between 1 and 5 reports in the literature search (Figure 2, Table 1). Kuwait is ranked second after SA for reporting *Cryptosporidium* infections (5 reports). In a Kuwaiti study that estimated the infectious and parasitic diseases mortality, there has been a steady decline in the number of deaths from infectious and parasitic diseases in Kuwait since 1975. This decrease in deaths has dropped from 758 in 1975 to 236 in 1983. However, when the researcher compared the death rate from infectious and parasitic diseases between Kuwait and selected developed countries, the study showed that, despite considerable improvement, the real rate of infectious and parasitic mortality in Kuwait remains very high compared to that in developed countries [81].

In Qatar and UAE, the reporting system for *Cryptosporidium* infection can be considered marginal, although they have rich economies indicated by per capita Gross National Income (GNI) [82]. The few reports that have been published from the GCC countries, with regard to parasitic infections, appear to give a false sense of security that these diarrhoeal parasitic pathogens may not be a serious problem in the region. GCC countries that neglect to screen or report the occurrence of cryptosporidiosis cases could be misinterpreted as having an absence or low prevalence of *Cryptosporidium* in those countries. Recent published data have highlighted the importance of monitoring and investigating intestinal parasites after several worldwide *Cryptosporidium* outbreaks.

Bahrain is the only country in the GCC region that does not have a published record for *Cryptosporidium* infections. In spite of reporting helminths and other protozoa in humans since 1995 [83], *Cryptosporidium* has not been considered or included in routine investigations of diarrhoeal infections. Bahrain has a relatively smaller economy than its oil-rich neighbours in the Arab Gulf. Over the years, Bahrain’s oil production has deteriorated dramatically, resulting in a high unemployment rate and poverty (11% of citizens), which may explain in part its neglected focus and research implementation of neglected diseases [84,85].

In about 70% of the reported studies in the GCC countries, *Cryptosporidium* has been linked to gastrointestinal symptoms, particularly diarrhoea in children under 5 years old (Table 1). In the Middle East, 76% (4348 volunteers) of military soldiers have reported at least one diarrhoeal episode [42]. In 45% of the cases, diarrhoea resulted in a median of 3 days of lost work productivity and a median of 2 days confinement to bed. Adverse effects of diarrhoea have caused 62% of the affected subjects to seek medical attention and subsequent intravenous rehydration from diarrhoeal complications [42]. In the GCC countries, other categories of adult patients (immunocompromised, Umrah people, and expatriates/immigrants) have also reported diarrhoea that had been caused by *Cryptosporidium* infection [39,49,50,65,66,68,70].

If this is indeed the situation with adult diarrhoeal cases, it would be expected that children under 5 years are more vulnerable to the adverse effects of diarrhoea from *Cryptosporidium* infection.

Two paediatric case reports as early as 1989 have linked *Cryptosporidium* infection to symptoms of severe diarrhoea, vomiting, and low-grade fever in children from Kuwait [86]. Over one third of the country’s infectious and parasitic deaths were reported as diarrhoeal deaths of infants and young children [81]. In Jeddah, the largest commercial city of Saudi Arabia, it was identified that 14.9% of school children have reported diarrhoea during the previous month in a study focusing on boys’ public schools (24 schools) that serve children aged 7–12 years. The main risk factor indicated in the analysis of the study was the number of children under the age of five living in the same household. Other risk factors associated with an increased risk of diarrhoea that was noted in the study are sewage spillage near the home, no drying for hands after washing, use of reusable cloths to dry dishes, and eating out after school hours [87]. In UAE, a survey of 500 parents with children under 5 years of age have reported that 87% of parents sought medical care for their children for the treatment of acute gastroenteritis within a three-month period, where 10% of those children required hospitalization with an average length of stay of 2.6 days due to complications of severe diarrhoea [88]. Asymptomatic children with cryptosporidiosis are considered to be carriers and act as important reservoirs for *Cryptosporidium* oocysts in the community [22].

In the Global Burden of Diseases (GBD), Injuries, and Risk Factors study, *Cryptosporidium* infection was the fifth leading cause of diarrhoeal mortality in children younger than 5 years, causing 48,300 deaths in 2016. According to the study, for every episode of cryptosporidial diarrhoea, there was an associated decrease in height-for-age, weight-for-height, and weight-for-age Z scores, which translated into an additional 7.85 million DALYs [11]. In North Africa and the Middle East, researchers have distributed the DALYs source in children under 5 due to *Cryptosporidium* infection into 40% wasting, 24% acute diarrhoea, 23% underweight, 9% stunting, and 3% protein energy malnutrition [11].

Paediatric diarrhoea has significant consequences on productivity and the financial impact on the livelihood of the affected families [44]. In the GCC countries, there has been a notable economic burden due to diarrhoea in children. For example, the total cost of hospitalization in Oman due to paediatric diarrhoea was estimated to be $539/child/3 days stay in hospital. For all outpatient and hospital settings in Oman, the total cost reached $1.8 million per year [89]. In the UAE, the average cost for medical care per paediatric diarrhoeal episode has been estimated to be $64 [88].

The lack of comprehensive studies on *Cryptosporidium* infection in paediatric diarrhoeal cases need to be strengthened in the GCC countries to reduce the economic burden associated with diarrhoeal diseases, to provide healthy children without long lasting adverse effects, and to reduce the transmission circle between family members and between families where the child is always the focus of *Cryptosporidium* infection.

Diagnosis and identification of *Cryptosporidium* infections in the GCC countries varies among the reports. The majority of them are based on the use of staining methods; however, occasionally confirmation of staining is combined with other sensitive methods like immune tests and PCR to make diagnosis (Table 1). The diagnostic method of choice for the detection and identification of *Cryptosporidium* usually varies according to the investigator’s goal as well as the available facilities and resources to make the diagnosis [90].

The prevalence of *Cryptosporidium* infection also varies among the GCC countries, with a prevalence ranging between 0.1 and 69.9%. The studies that have depended on combined stains and immune tests authors noted a wide range of prevalence between 0.1 and 69.9%, while studies that have used PCR methods to confirm *Cryptosporidium* prevalence ranged between 1.7 and 19.4%. Only one study has reported a high prevalence of 94% by the authors, who used PCR to analyse previously confirmed positive samples via staining [50] (Table 1).

Only eight out of 28 studies (28.6%) from GCC countries have further processed their isolates by molecular analysis to verify the geno-/subtyping of *Cryptosporidium* spp.. The molecular methods used in these studies varied between arbitrarily primed PCR, qPCR, sequencing, and PCR-RFLP, where PCR-RFLP was the most commonly used technique to the identify the *Cryptosporidium* spp. and subtype (Table 2).

PCR methods are well established techniques that are used to detect *Cryptosporidium* DNA in samples with accuracy, sensitivity, and specificity over traditional staining methods. Quantitative PCR (qPCR) is known to be the most accurate amongst the PCR methods due to a decreased risk of sample contamination; early reporting of results, particularly during outbreak investigations; and with the detection and quantitation of the target nucleotide sequences down to one or a few copies per samples [90]. The majority of the GCC studies used PCR-RFLP to detect *Cryptosporidium* spp., probably due to the lower costs associated with this highly accurate technique.

We have concluded that the studies that used PCR methods had the most realistic prevalence and burden numbers of cryptosporidiosis in the GCC countries (1.7–19.4%). Other factors must be considered that can affect the prevalence of *Cryptosporidium* in these studies. For example, differences in method, number and type of diagnostic method used, number of selected samples for the study, target population, aim of the study, state of the population’s health, symptomatology, and expertise of investigators.

*C. parvum*, *C. hominis*, *C. meleagridis*, and *C. muris* have been the identified species that infect humans in the GCC countries [45,46,49,50,67,70,78]. Distribution of different *Cryptosporidium* genotypes in human populations can be considered an indication of the differences in infection sources [91].

*C. parvum* has been reported to be the dominant species in isolates from the GCC countries. In Kuwait, *C. parvum* has been identified as the predominant causative species of cryptosporidiosis in children [49,50]. In Qatar, it was the principal species as well in the Qatari children and expatriates [45,46]. Saudi Arabian children from Gizan and Maddina were also dominantly infected with *C. parvum* [67].

*C. parvum* is a species that infects a broad range of mammals and is considered one of the major zoonotic disease problems [92]. Its dominance in the GCC countries indicates that there is an animal-to-human transmission, particularly when subtyping outcomes are considered.

From subtyping data of *Cryptosporidium* infections in the GCC countries, *C. parvum* IId has been shown to be the predominant subtype family in most of the GCC countries (Table 2). The IId subtype has been referred to as the major zoonotic subtype family in Europe, Asia, Egypt, and Australia [93,94,95,96,97,98]. Its distribution has been associated with the domestication of goats, sheep, calves, horses, donkeys, and takins [99]. According to Qatari and Kuwaiti paediatric diarrhoeal studies that have investigated the risk factors associated with *Cryptosporidium* infection, there has been limited, if indeed any, contact with farm animals when investigators were considering the source of initial infection [46,49]. On the other hand, the frequent reporting of the IId subtype family in the GCC countries suggests the potential occurrence of zoonotic transmission of *C. parvum*.

The Qatari studies have indicated that there is a predominance of the IId subtype family in its hospitalized children and immigrants, and suggested that *Cryptosporidium* contamination from foodborne transmission or person-to-person contact, but there is no indication that the source of infection could also be from contaminated water or contact to animals [45,46]. None of the Qatari studies reported prevalence or occurrence of *Cryptosporidium* spp. in local animals or drinking water. One study from Kuwait has indicated that nine of the paediatric cryptosporidiosis cases had direct contact with animals but did not demonstrate any significant association between the risks of infection from those animals [49]. Another study in Kuwait has investigated 47 sheep and goat farms and found a predominance of the *C. parvum* IId subtype family in two-thirds of the infected animals [95]. In Saudi Arabia, *Cryptosporidium* has been detected in camels, sheep, and goats, but there has been no further molecular identification of these species and subtypes [100].

More research is needed in the Gulf region to confirm if animal contact is a major source of infection. The prevalence of the *Cryptosporidium* needs to be investigated in the animal population. In addition, the authors of this review have speculated that if the elderly populations were included in the Gulf research studies, there may be a significant correlation between the *Cryptosporidium* positive cases and contact with animals, particularly in Arab falconers and those who enjoy breeding and riding camels (see details in the next chapter).

*C. hominis* is a species mainly restricted to humans (anthroponotic transmission) despite it has been recently reported in young calves [101]. It has been reported to be the predominant species in children from Makkah, Saudi Arabia [70]. Other studies have noted its occurrence in a few number of cases from Qatari immigrants (1) and hospitalized children (4) [45,46], Kuwaiti symptomatic children (15) [49,50], and Saudi Arabian children (13) [67] (Table 1). Person-to-person contact is also a plausible way to contract cryptosporidiosis in the GCC countries; however, it appears to only represent a very small percentage of cases in the available literature.

*C. meleagridis* and *C. muris* have been the least reported species in the GCC countries. Qatar and SA are the only countries that reported these species from their isolates. *C. meleagridis* has been described within mixed infections of *C. parvum* in two Qatari reports (children and immigrants) [45,46] and as a single species infection in asymptomatic Saudi children [78], whereas its transmission has not been clarified in any of those studies. *C. meleagridis* primarily infects birds and mammals and is considered the third most common cause of cryptosporidiosis in humans [102], despite it frequently being reported in particular populations of Thailand, Peru, and Japan [9,103]. The Qatari cases with *C. meleagridis* infection seem to be linked to travel to endemic areas or countries, or were infected from people coming from endemic areas or contact with birds, e.g., falcons.

A single *C. muris* case has been reported in one Saudi child; however, the conclusions are marginal since the authors reported PCR technical difficulties with processing the *C. muris* DNA. Further, this particular isolate was the only species that was withheld from the gel electrophoresis during their PCR-RFLP analysis [78].

Zoonotic and anthroponotic transmissions of oocysts are known pathways for *Cryptosporidium* infection in the Gulf population. It is essential that Gulf governments, public health authorities, and investigators consider publishing more investigations on cryptosporidiosis in animals and symptomatic individuals who have had direct contact with those animals. It would be worthy to combine human and animal investigations in one study for the detection of *Cryptosporidium* that uses molecular analysis to verify the genotype/subtype prevalence in human and animal populations.

## 4. Possible Risk Factors in the GCC Countries Associated with the Prevalence of Cryptosporidiosis

Poor water quality, animal contact, overcrowded living conditions, household diarrhoea, and open defaecation have been identified as significant risk factors for *Cryptosporidium* infection in low- and middle-income countries [29]. Countries that have been identified as “poor income countries” can suffer additional risk factors that double the predisposition for cryptosporidiosis. These risk factors include inadequate water supply, water crises, unclean water, poverty, illiteracy, social unrest, climate change, political conflict, and underdevelopment, which can create dramatic consequences in the poorest members of this population [19,20,21].

Due to the high-income status of the GCC countries, the risk factors for *Cryptosporidium* infection and other infectious diseases are notably lower than those in the “poor income” category. Collectively, the Gulf reports have only addressed one major risk factor (expatriates) but neglected to specify other epidemiological factors that may contribute to *Cryptosporidium* infection in the region. The most putative important risk factors for cryptosporidiosis in the GCC countries will be presented in the following sections.

### 4.1. Cryptosporidium Contaminating Water Resources in the GCC Countries

The GCC countries are considered the poorest region in the world in its water resources. This is due to their geological location and climate. They are characterized by their arid environment (hot and dry) with irregular and infrequent rainfall, high evaporation rate, and scarcity of renewable water resources [35,104]. Arid regions have a higher correlation between available water resources and public health problems [36], which can consequently have a negative impact on the social and economic development in the region.

The GCC countries depend mainly on water desalination, which is an expensive process that removes salts and minerals from seawater and brackish water [37]. There is almost no surface water either in the GCC countries [32,105]. Due to the rapid expansion of the population, lifestyle changes have occurred with the urbanization and reclamation of agricultural areas, where valuable groundwater is extracted to satisfy the demand for water [36,104]. Fortunately, the desalinated seawater can provide an unlimited supply of drinking water, although it does come with a risk when it is inadequately produced and contaminated or if the water treatment systems fails [38].

Prior to pumping desalinated water into the distribution network, the water is chemically treated. In Jeddah, Saudi Arabia, the drinking water is only distributed to properties once or twice per week. The processed water is then stored in private underground tanks for two days. Afterwards, the stored water reaches the distribution facilities, where it is pumped to roof tanks on homes and businesses to be available when needed [87]. In many areas of Jeddah, the domestic wastewater system uses a cesspool, which runs next to the underground water storage tanks. The long-term use of a cesspool system has caused a rapid rise in the underground water table. This has led to contamination of potable water stored in the underground tanks [106,107]. In the western provinces of SA, the use of conventional on-site sewage systems is the exclusive pathway to dispose sewage. Under ideal conditions, the waste effluent is assimilated and treated within the topsoil that is directly adjacent to the cesspool, without regulation or implementation, to ensure there is enough separation between the bottom of the cesspool and the water table [108]. It has been confirmed that the fate and movement of the chemical constituents (nitrates) and bacterial contamination from this septic/cesspool effluent mixes into the shallow groundwater, private shallow and deep wells, and dump stations [108,109,110].

It is recognized that the on-site sewage disposal systems have contaminated the drinking water sources and subsequently caused health problems in the Gulf region. If chemical and bacterial contamination is present in the drinking water, it is expected to have parasitic contamination as well; however, this parameter is under recognized in the GCC countries.

Although *Cryptosporidium* has been frequently detected in faecal samples of local inhabitants in the GCC countries (SA [66,76,77], Kuwait [50], UAE [80], Qatar [46], and Oman [64]), they have little published data regarding the occurrence of *Cryptosporidium* in the Gulf water supply. However, six studies in SA, UAE, and Kuwait have investigated *Cryptosporidium* in selected water resources in the GCC (Table 3), with interesting outcomes.

It is remarkable that *Cryptosporidium* was present in almost all water resources from the GCC countries, which included desalinated water, underground water, bottled water, swimming pools, irrigation water, and chlorinated water from sewage treatment plants [40,49,111,112,113,114].

In the SA city of Al-Taif, *Cryptosporidium* has been identified in 8% of desalinated water samples [40]. In Makkah, another SA city located next to Al-Taif, the presence of *Cryptosporidium* infection among its inhabitants has been suspected to originate from contamination from the local desalinated water system. Due to the similarity and construction of the two desalination water systems, this has led investigators to suspect the desalination water system as the most plausible source of *Cryptosporidium* infection in Makkah [40,70].

The high prevalence of *Cryptosporidium* in Kuwait has been linked to the winter desert camping areas, where large numbers of overhead water storage tanks are used to store potable water. Water tanker trucks transport this desalinated water to these camping places. It is very interesting that the *Cryptosporidium* subtyping result from the contaminated tank water has been identified as *C. parvum* subtype IIa, and that five members of the same family using this water source at the camp were also infected with the same subtype [49]. This has provided a direct link to contaminated desalinated water as a potential source of *Cryptosporidium* infection. Moreover, the contamination of water with oocysts has probably occurred at the end of the water treatment process during distribution [49].

It has been reported that about 7.3% of underground waters (wells) are contaminated with *Cryptosporidium* in Al-Taif [40]. The protected wells were previously found to be contaminated with faecal matter [115]. It is not be surprising if unprotected wells are contaminated from a variety of sources, such as wastewater effluent, overland flow from manure piles, as well as domestic or wild animal grazing. Fossil groundwater covers about two-thirds of the Arabian Peninsula, and it is the main source of water in the GCC countries [116]. Ground water pollution in the GCC countries has been caused mainly due to over-pumping from wells. However, there are other factors that have contributed to ground water pollution, such as irrigation returns, seawater intrusions, liquid effluents from septic tanks, and agricultural chemicals. These factors have led to the abandonment of many water wells in the GCC countries [117]. Water well pollution highlights the necessity of higher water-protection legislation and conservation to ensure the protection of water supply for all inhabitants [118].

Bottled water in Tabuk, Jeddah, and Mekkah in SA has been reported to be contaminated with *Cryptosporidium* using modified Ziehl Neelsen (MZN) as a diagnostic method [111,112]. In these two studies, the authors have not given clear details regarding the water samples used in their investigations and they published ambiguous results concerning the bottled water contamination. In comparison, another study from Al-Taif, using nested PCR and five brands of bottled water (domestic and imported), has reported all samples to be free from *Cryptosporidium* oocysts [40].

The microbiological quality of bottled water has been the focus in UAE since 1999. Although authors have mentioned that the presence of bacteria in bottled water can act as an indicator for the possible presence of *Cryptosporidium*, there has been no established method yet to screen the bottled water for this protozoan parasite in the GCC region [119].

As mentioned from some of the literature, the quality of bottled water can vary between brands. Researchers have speculated that it might not be any safer than tap water, unless it is distilled or pasteurized to ensure complete disinfection. The source of the bottled water is also very important, especially if it is collected from a surface water source (e.g., a stream) and it may be more likely to contain *Cryptosporidium* and other microorganisms than bottled water derived from a ground water source (e.g., a well). Therefore, it is important for companies that sell bottled water to also list the water source on the product label [120,121].

In one study, indoor and outdoor swimming pools from five Emirati schools were found to be contaminated with an average concentration of *Cryptosporidium* between 1 and 15 oocysts/L. The ages of the swimmers were between 3 and 14 years old, who attended 1–3 swimming classes per week [113]. Due to the hot weather in the GCC countries, many swimming pools are available at schools, hotels, parks, and residential areas that are frequently used by many individuals from various age groups. Formed faecal incidents (poop) pose a risk for the spread of infectious disease, including parasitic protozoa [122]. The CDC’s Healthy Swimming Program has indicated that *Escherichia coli*, a faecal indicator, has been detected in 93 (58%) of the swimming pools samples, and further explains the necessity of regular monitoring for chlorine-resistant *Cryptosporidium* oocysts [123]. Detection can signify that swimmers have introduced contaminated faecal material into swimming pools either when it washes off a swimmer’s body or by release of a formed (or diarrhoeal) faecal incident into the water.

The overuse of swimming pools can significantly compromise the effectiveness of proper cleaning and decontamination efforts. The risk of contamination for *Cryptosporidium* in swimming pools is therefore estimated to be very high in spite of use of filtration and chlorination as a cleaning and sanitization method [113].

The usage of chlorine as a water disinfectant is known to be effective against many microorganisms; however, *Cryptosporidium* oocysts are resistant to the effects of chlorine [124] and various environmental stresses, such as extreme temperature variations [40]. The oocysts are small (5 µm) and have a low infectious dose (1–10 oocysts), and reportedly has the ability to maintain viability in water longer than 6–12 months or longer with the capability to cause epidemics, even after the consumption of purified drinking water [2,125,126].

In the GCC countries, bacterial and fungal indices are routinely tested in different water resources [113,127,128]; however, only scientific institutions care to identify the absence or presence of *Cryptosporidium* oocysts in water samples.

The Dubai municipality environmental safety inspectors, who send samples to the central laboratories, do not consider the presence of *Cryptosporidium* oocysts in swimming pool water as an indicator of its quality, while instead mainly focusing on monitoring for bacterial indicators [113]. The National, the leading English news service of the UAE, has warned against the failure to keep UAE pools clean due to insufficient disinfection and expressed concerns for infectious disease in swimming pools, including parasites that are known to cause severe diarrhoea amongst children. They have reported that when humans become infected with *Cryptosporidium*, they can act as carriers and release its chlorine-tolerant-oocysts into the swimming pools, and suggested that UV irradiation be applied instead of ineffective chlorine for the disinfection of swimming pools [129]. It remains uncertain, however, whether and in what extent UV treatment has a real impact on *Cryptosporidium* during the water treatment process. Only public and private action on such warnings in all GCC countries can help protect the most vulnerable populations (e.g., children and immunocompromised individuals) from becoming infected with *Cryptosporidium*.

*Cryptosporidium* oocysts have been detected in 94.4% of the irrigation water used in public parks in UAE [114]. *Cryptosporidium* oocysts have also been found in chlorinated water samples, as well as effluent samples collected from sewage treatment plants [114]—an indication that the water treatment systems (wastewater disinfection) have failed to eradicate the transmissible stages of *Cryptosporidium* in the water treatment process. In the UAE, it is not routine to test for the presence of *Cryptosporidium* oocysts in recreational water and reclaimed wastewater, while bacteriological (total and faecal coliforms) indices are the only biological parameters used to assess their water quality [114,130].

The GCC countries produce a large amount of wastewater with an average of 2.853 Bm3/year [104]. This wastewater has been reported to contain a wide range of pathogens, including parasites, viruses, and bacteria [131,132,133], and represents a real challenge when designing conventional treatment plants that can meet the health guidelines of the Environmental Protection Agency [131]. Status of average renewable water resources per capita in the GCC countries has already shown a warning sign, and due to the water crisis conditions they often use improperly disinfected wastewater for irrigation [134].

Water contamination with *Cryptosporidium* is an under-recognised and under-investigated problem in the GCC countries, and probably one of the main sources of diarrhoeal diseases in the region. Political and social support is required to include *Cryptosporidium* and other protozoan parasites in the testing framework for water quality and reuse of treated water. A lack of water surveillance systems has been noted in the GCC countries. Water research that includes analyses of the *Cryptosporidium* genotypes and subtypes will help strengthen the available information about the extent of this pathogenic parasite and its main sources. It would be also effective if the Gulf governments consider funding infrastructural projects to efficiently treat water using good installation facilities and proper pre-treatment of chemicals in the process design.

### 4.2. Animals and Birds Invading Sports Events in the GCC Countries

In the GCC countries, only a small number of studies have been performed on the presence of *Cryptosporidium* in different animals. However, nine of the published studies have emphasized the concept that animals can be a significant source of *Cryptosporidium* infection in the Gulf human population. Whether they are used domestically or ridden during sporting events or leisure activities, various animals and birds (sheep, goats, calves, camels, lambs, Arabian oryx, falcons, and stone curlews) have tested positive for *Cryptosporidium* infection in the GCC region (Table 4).

On a well-managed Omani farm that maintains closed herds of goats, sheep, cows, and buffalo, with regular vaccinations, a severe cryptosporidiosis outbreak has been reported in goats [137]. Massive catarrhal enteritis with markedly enlarged mesenteric lymph nodes have been observed in post-mortem goats due to an invasion of large numbers of *Cryptosporidium* oocysts. Another diarrhoeal outbreak in the UAE that has occurred was in juvenile stone curlews [141]. Although the owner maintained a good breeding system for the stone curlews, they all became infected with *Cryptosporidium*. Numerous endogenous cryptosporidial stages were confirmed in their histopathological sections. Despite intense supportive care, both outbreaks have resulted in a high mortality in animals (238 kid goats and 14 adult animals died) and birds (19 stone curlews died). *C. parvum* has been determined to be the main species that caused both outbreaks; however, both studies failed to recognize the main source of infection [137,141].

Domestic livestock, especially goats and sheep, are widely raised for meat production in the GCC countries [142]. In SA, 22.2% of sheep and 10.3% of goats have been reported to be infected with *Cryptosporidium* on three farms located in Riyadh [100]. In Kuwait, likewise a wide range of domestic animals (goats, sheep, lambs, and newborn calves) have been screened for the presence of *Cryptosporidium* infection [95,135,136], where sheep and goats constitute the majority of its livestock. These animals have the ability to adapt to the arid climatic conditions (hot/dry season and wet/cool season). *Cryptosporidium* has been reported to be prevalent in 11.4% and 7.2% of sheep and goats, respectively. *C. parvum* has been noted to be the dominant species responsible for the high frequency of caprine and ovine cryptosporidiosis, and infection is usually associated with a large-size herd (overcrowding in a closed housing system), poor hygiene, and poor management practices on the Kuwaiti farms [95].

Many animals were imported into Kuwait, particularly cattle, to re-establish the animal industry after the end of the Iraqi invasion. During the first three weeks of life, calves from eight dairy farms in Sulaibyia have suffered from severe diarrhoea, being unresponsive to antibiotics, which ended with a calf mortality of 40% and morbidity of 20–60%. The authors have reported that *Cryptosporidium* was the main attributor to the diarrhoeal aetiology in the neonate calf deaths [136]. Housing pens with dirt floors, accumulated manure with no regular removal, early separation from dams, and an intensive system (large number of animals raised on limited space of land) have all been cited factors in studies that might help ease the transmission of *Cryptosporidium* oocysts in calves [135,136]. Infected calves are known to excrete large numbers of *Cryptosporidium* oocysts that might reach millions [143] and therefore likely able to rapidly transmit the infection among herds.

It deserves mentioning that the sequence analysis of the *C. parvum* spp. in ruminants isolates (IIdA20G1 and IIaA15G2R1) from Kuwait [95] have been previously documented as dominant subtypes in the infected Kuwaiti children [50], suggesting that domestic animals can be potential zoonotic reservoirs for cryptosporidiosis and a source of cross contamination in the environment.

Similar to the situation in Kuwait above for cattle imports, cattle were flown into Qatar to raise supplies of milk in the midst of a country blockade led by Saudi Arabia. According to the BBC news, the dairy cows (Holstein) came from Germany—the first of about 4000 cattle to be imported was first imported into Qatar. Air, sea, and land restrictions have created turmoil in Qatar, which is dependent on imports to meet the basic needs of its 2.7 million inhabitants. Several thousand cattle were later imported from other countries. It remains unknown what epidemiological significance such animals will have for the distribution of cryptosporidial oocysts in the country.

Animals, whether enjoyed during sporting events or for riding for pleasure, such as camels and captive birds (falcons and stone curlews), have become the focus of *Cryptosporidium* research in SA and UAE countries. In the SA city of Riyadh, *Cryptosporidium* has been ranked first among the microorganisms (*Escherichia coli*, Corona, and Rota virus) that can cause diarrhoea in 15% of the symptomatic camel calves from that area [138]. Samples of camel faeces in the same city have been noted to be highly infected with *Cryptosporidium* oocysts (22.4%) compared to goats and sheep that were screened using MZN and ELISA methods in another study [100]. Camels are the principal domestic animal in SA and are used as a source of meat and milk. They are likewise used for racing sports and transportation [144]. In Kuwait, camels are often utilized for pleasure rides beside families who are camping in the desert. Although they are reported to be infected with *Cryptosporidium* since 1991 [145], they were excluded as a possible source of *Cryptosporidium* infection in Kuwaiti residents who had been infected during a camping incident [49]. In UAE, researchers have tested for the presence of antibodies against many infectious diseases, including protozoa, and these have been reported in their racing camels [146].

Camel racing in the Gulf region has returned to the height of its cultural revival [147] due to its adaptation to life in the hot and arid regions [148]. Although Gulf camels have been known as carriers for many zoonotic parasites [149], since 1994, screening for *Cryptosporidium* and other protozoa has been probably ignored in camels and the people in close contact with them.

Zoonotic pathogens carried by camels are a current future risk to public health [150]. The role of camels in the transmission, distribution, and maintenance of *Cryptosporidium* in the GCC countries should be investigated by governmental authorities and researchers alike, especially in light of the increased use as an increasing source of protein and a sporting gain.

Captive bred birds (e.g., falcons and stone curlews) are a popular hobby for Arab falconers. In UAE, two falcons have been identified with cryptosporidiosis during a routine health check. Their faecal samples and lung tissues tested positive for *C. parvum*. In that study, the two falcons were totally asymptomatic for any intestinal or respiratory signs [140]. Conversely, it was reported that *C. parvum* caused severe symptomatic manifestations (catarrhal enteritis) with a high mortality rate in captive stone curlews in Dubai [141].

The UAE has no routine testing for the presence of *Cryptosporidium* spp. in birds, owing to the lack of regional specialized laboratories. Even though both falcons were bred in the UAE, unfortunately the authors of the study were unable to identify the source of the *C. parvum* infection and failed to check their owner, “the first suspect”, for the possibility of having cryptosporidiosis [140].

### 4.3. Expatriate Labourers in the GCC Countries

A greater risk for *Cryptosporidium* infection has been linked to a low socioeconomic status [19,22] and travel to developing countries, where poor water treatment and lack of food sanitation are prevalent [151].

Gulf researchers often use terms like expatriates, immigrants, or guests for people who come to GCC countries seeking a better financial situation. Sustained economic stability and rapid socioeconomic developments have attracted expatriate workers with mass influx into the GCC countries. These multinational guest workers are mainly from developing countries with a low socioeconomic status [47]. A factor that has long been associated with the transmission of parasitic diseases and is one of the main focuses of research in the GCC countries.

During the pre-employment stage (at the country of origin), expatriate workers are screened for the presence of ova and intestinal worms via stool analysis and culture. Although the expatriates must be free of contagious and infectious diseases (HIV, HCV, and HBV) to be allowed entrance into the GCC countries, *Cryptosporidium*, a known pathogenic protozoan, is generally not included on the medical examination list of investigations [152].

Various studies in different GCC countries (SA, Qatar, UAE, Kuwait, and Oman) have monitored for intestinal parasites among expatriates. It has been reported that the majority of these workers, including food handlers, housemaids, domestic helpers, babysitters, drivers, and private cooks, have tested positive for parasitic infections in the Arabian Gulf [53,63,79,153,154,155,156,157,158].

The prevalence of *Cryptosporidium* has been investigated among expatriates (adults and children categories) from Oman, Qatar, SA, and UAE (Table 1), who have mainly originated from developing countries (Afghanistan, Bangladesh, Ethiopia, India, Indonesia, Nepal, Pakistan, Philippine, Sri Lanka, Turkey, Egypt, and Jordan). These countries are known to be endemic with many infectious diseases, including parasitic diseases. Moreover, many risk factors have been reported to be associated with expatriate workers that predispose themselves to cryptosporidiosis [45,47,48,76].

In the UAE, expatriate workers mainly originate from Asian, African, and Arabic countries, where the majority of them are from Asia. These migrant workers from Asia have the highest prevalence rate of *Cryptosporidium* infection among the guest worker population. In their home countries, they live in rural settings under crowded conditions and have poor sanitation, predisposing them to infectious diseases. Migrant workers are often required to stay in similar living conditions in their CGG work destinations, where they may have to live in labour accommodations and share the same bedroom (with ≥6 persons) and toilet (with >5 persons) with many people [47,48].

During the Umrah season in Makkah, SA, there is crowding of a hundred thousand Muslims from different nationalities with close contact and congestions between the pilgrims and local inhabitants. The overcrowding and overcapacity of available accommodations has been noted as an important risk factors for *Cryptosporidium* infection during the Umrah season [60].

In Qatar, expatriates from Western and Eastern Asia as well as North and sub-Saharan Africa have been examined for risk factors and the prevalence of cryptosporidiosis. In the GCC countries, many Asian individuals (Indian and Filipinos) who hold jobs, such as housemaids, builders, mechanics, cleaners, masons, and carpenters (blue collars), have tested positive for *Cryptosporidium* infection. At the country of origin, expatriates who have been infected with *Cryptosporidium* had many of the risk factors associated with parasitic infection, including a low education level (elementary school only), low home index, low monthly income, and those who were accustomed to using pit latrines [45]. Children of expatriates from the Middle East, Asia, Africa, and the local Qatari population have been examined for intestinal parasites, whereas *C. parvum* was the most common incidental parasite affecting 14.7% of cases. Surprisingly, Qatari nationals had the highest number of parasitic infections from any other group tested in spite of fewer reported cases in the local Qatari population when compared to the expatriate groups (168 versus 412) [46].

In Oman, it has been reported that many of expatriate Indian food handlers were infected with multiple intestinal parasites, including *Cryptosporidium.* The authors have stressed in their report that it is necessary to screen food handlers for parasitic infection using different diagnostic methods, especially before these individuals are allowed to work in restaurants, hotels, factories, and private homes [63]. Poor personal hygiene among expatriate food handlers has been emphasized in the literature to be a significant contributor to foodborne outbreaks [159].

In the context of good hygiene and safety in food handling, multiple risk factors linked to expatriates in the GCC region are noted to promote cryptosporidiosis, which is a threat to public health. Social marginalization in the form of low socioeconomic status, low living standards, low education, overcrowding, and unhygienic practices (lack of personal hygiene and/or non-practicing of proper hand washing before eating or handling food) are high risk factors for *Cryptosporidium* infection.

Symptomatic expatriates (mainly food handlers and housemaids) have a greater potential to inadvertently introduce contaminated faecal material into the food industry when working with food and food processing facilitates and equipment (indirect pathway) or by infecting another person in the household or business of their employer (direct pathway). If this happens, *Cryptosporidium* oocysts will circulate in the community (locals and expatriates) until this outbreak cycle can be halted. *Cryptosporidium* oocysts are well known to be environmentally stable, allowing them to be highly infective within vulnerable groups (e.g., children and immunocompromised individuals). Accordingly, it is crucial to increase the health awareness among expatriates (particularly food handlers, housemaids, and babysitters) about different transmission routes of *Cryptosporidium* and the important requirement for its prevention and control.

It is interesting to note that the prevalence of *Cryptosporidium* and other intestinal parasites in expatriates has been reported to be lower in Gulf studies when compared to the population of their home countries [46,63,160].

There has not been a single study that compares the prevalence of *Cryptosporidium* infection between expatriates who have recently entered a GCC country and those who spent a long period of time there. The discussion table comes with a significant point about the source of infection. Either expatriates come from their home country with the infection, or they have been infected in the country of their employment.

Further studies on the health status of Gulf natives are therefore urgently required to get a true estimate of the source of *Cryptosporidium* prevalence and finally answer the following questions: Who is infecting whom? Do foreigners import *Cryptosporidium* oocysts and other infections to the Gulf, or are the Gulf locals actually infecting the foreigners?

More research is needed to clarify *Cryptosporidium* transmission cycle in the GCC countries.

### 4.4. Food as a Vehicle of Foodborne Cryptosporidiosis in the GCC Countries

The high economic position of the GGC countries has established itself among the more food-secure and high-income countries in the world. This situation has created significant pressure on the available natural resources and food production capability in the region. The six GCC states have limited control over their food sources and production capabilities with limited sustainability due environmental challenges [35]. Additionally, the population of the GCC countries has significantly expanded due to the invitation of large numbers of expatriate guest workers who are needed to help industrialize and urbanize these affluent oil producing countries.

In the GCC region, many efforts have been made to transform the arid deserts into more habitable areas by using progressive desalinization and desertification processes. Moreover, many challenges must still be overcome to tackle this difficult environment (high temperature and scarce water), where its soils are sandy, fragile, and poorly enriched with organic matter [161,162].

Agricultural land in the GCC countries accounts for 19.5% of total land area available, whereas only 1–2% is actually arable (cropland regularly ploughed or tilled) [163]. Therefore, the GCC countries are forced to rely on imported food to meet their high demands [35,52].

Approximately 90% of the GCC’s food and drinks are imported. Annually they import around 33 million tons of foods with expectations to increase in the future to satisfy their expanding economies [164]. Therefore, great emphasis is placed on food safety and security for all imported foods into the GCC countries, including legislation and guidelines to safeguard the quality of the imported food [37,165]. However, their traditional food safety systems have not properly developed to identify potential problems (e.g., infectious disease and parasites) in the food supply before they occur, but rather they are organized to respond to foodborne outbreaks [166].

Contaminated food and drinks with *Cryptosporidium* oocysts and other pathogenic microorganisms are important routes for foodborne outbreaks of cryptosporidiosis far and wide. The catering and food service industries use many high-risk food materials (vegetables, fruits, shellfish, and meat) that are potentially contaminated with *Cryptosporidium* and have been responsible for occasional outbreaks in the past [18].

The GCC countries, along with other Middle East countries, have been classified to have the third-highest estimated burden of foodborne diseases per population, directly behind the African and South-East Asian regions. Foodborne pathogens in these regions have caused illnesses in 100 million people per year, and 32 million of those affected are children under five years [167]. Gastrointestinal infections that are frequently seen in the Gulf region are primarily caused by *Salmonella* spp., followed by *Shigella* spp. and other pathogens like hepatitis A virus and parasites [166,168]. Consumption of unpasteurized dairy products and commercial meat products have been implicated in foodborne diseases in Kuwait, Oman, and SA [165,169]. In Jeddah, SA, there has been a rapid increase in the number of fast food businesses owned by immigrants from developing countries who have not had adequate training in food hygiene. Fast food dishes have a great potential for food contamination due to undercooked meat that does not reach the criterial temperatures to kill microorganisms [87,170].

There are scattered reports about the role of bacteria and viruses as causative agents of foodborne diseases throughout the GCC region. Often, parasites, including *Cryptosporidium*, are the causative agents in foodborne diarrhoea; however, the actual available reports on diarrhoeal cases in the Arabian Gulf are scarce or non-existent.

Only one study in Qassim, SA, has investigated the different types of leafy vegetables (green onion, red radish, garden rocket, lettuce, and parsley) for the presence of parasites. The authors reported that all vegetables tested in the study had been contaminated with a variety of parasites, such as *Giardia*, *Balantidium coli*, *Entamoeba*, *Cryptosporidium*, *Trichuris, Enterobius*, and *Taenia* [171]. Other foodborne outbreaks have been documented in SA [172]. However, microbiological surveillance has been performed in the 31 reported foodborne outbreaks, while only *Salmonella* spp. and *Staphylococcus aureus* were the identified pathogens from outbreaks. Moreover, the authors declared that many foodborne outbreaks occur every year in the Kingdom of SA [172]; however, *Cryptosporidium* and other foodborne parasites have been nevertheless excluded from such investigations.

The GCC Ministerial Committee for Food Safety has established joint legislation and regulations on food safety based upon the certainty that imported foods may represent human health and environmental safety challenges. The food safety guidelines represent health certificates forums, technical regulations, and standards that list food categories and their certification requirements. The technical regulations emphasize the microbiological criteria and the general safety standards for contaminants and toxins [173]. Regrettably, the guidelines do not specify any regulations or laws concerning food safety from parasitic contamination, which have caused foodborne outbreaks such as cryptosporidiosis.

It is important to note that imported food could be contaminated with *Cryptosporidium* oocysts (a) from the country of origin due to contamination from animal or human faeces in the water or soil sources used to produce the food, or infected individuals that grow and store the food; (b) from infected individuals transporting the food on the way to the designated country; or (c) from within the destination country via infected food handlers or businesses that store the imported food in improper conditions or washing and preparing food with contaminated water.

GCC countries must apply well-developed strategies for prevention and control of foodborne cryptosporidiosis. The food security strategies must include surveillance systems in the health care system and food industry that monitor for the presence of *Cryptosporidium* oocysts. In addition, they must establish an epidemiological information system with local governmental authorities that also partners with applied researchers towards the advancement of technologies that can effectively detect and disinfect oocysts in food and water supply. There are needs to be a modification of current regulatory standards that specifically includes parasitic contamination in imported food and educational programs made available to food handlers in order to further reduce the risk and the incidence of foodborne illnesses, such as *Cryptosporidium* infection.

### 4.5. Airborne Transmission of Cryptosporidium under Arid Climate Conditions

The miniscule size of *Cryptosporidium* oocysts has the capability to disseminate across the air, where they could be inhaled and cause infection in humans and animals [174]. Inhalation of oocysts from contaminated air can infect the respiratory tract and manifest respiratory symptoms [175,176,177]. *Cryptosporidium* oocysts have been observed in 60% of the investigated air samples in Mexico [174]. Direct contamination with faecal material because of the lack of sanitary infrastructure results in a greater dispersion of soil via airborne dust during dry season, particularly in those places where people are exposed to large amounts of outdoor dust [174].

The GCC countries are characterized by arid climatic conditions (long, dry, hot summers and short, relatively warm winters) [49,95,113]. Weather conditions, such as heat, wind, and a lack of rainfall, have significantly contributed to dust and the formation of the GCC countries’ regional climate [178]. Therefore, the Gulf population has a higher exposure to large amounts of outdoor dust, which puts them at risk for *Cryptosporidium* infection from contaminated air particles; more so if they have close contact with infected livestock.

It has been reported in the epidemiology of cryptosporidiosis that respiratory aerosol droplets from infected individuals can be one of the crucial factors in the transmission, rapid spread, and continuous circulation of *Cryptosporidium* oocysts. Evidence has suggested that oocysts can be transmitted via respiratory secretions as well as through the more common faecal–oral route [177].

It has been documented that wind can increase the spread of viruses in the saliva and respiratory droplets when someone coughs or sneezes. Studies have demonstrated that airborne particles from sneezes can travel up to 6 m in 1.6 s with an accelerated dispersion rate [179]. The same scenario also could occur with respiratory droplets from individuals infected with *Cryptosporidium* oocysts. It has been shown that *Cryptosporidium* oocysts are able to infect epithelial organoids derived from human lungs and are successfully able to complete their lifecycle [180]. The risk of illness for *Cryptosporidium* oocyst air inhalation has been found to be very high and has shown to reach above the safety guidelines of its presence in water (1 × 10^−4^) [174].

With or without symptoms, *Cryptosporidium* oocysts are involved in the respiratory tracts of avian and some mammals, which includes a small number of human cases [177]. All of the published research studies from the GCC countries have not included or excluded questions regarding respiratory symptoms in the diagnosis. However, respiratory *Cryptosporidium* infections have been reported to occur in immunocompetent children with enteric cryptosporidiosis, individuals with an unexplained cough, and in immunocompetent adults with tuberculosis from Uganda [175,176]. It is worthy to stress that 35% of children with intestinal cryptosporidiosis and cough had *Cryptosporidium* DNA in their respiratory secretions [175], which validates the potential for *Cryptosporidium* to be transmitted by cough, sneeze, and expectoration from those who have cryptosporidial infections and diarrhoea.

In the UAE, two asymptomatic captive falcons were identified to have cryptosporidiosis and tested positive for *C. parvum* in their lung tissues by molecular analysis. In addition, the main endoscopic findings from the cases indicated an infectious process in the ostia, caudal lung field, and caudal thoracic air sacs with an accumulation of inflammatory cells. Acid-fast positive cryptosporidial oocysts was identified as the cause of the infections in the report [140]. Although, the *Cryptosporidium* infection in the falcon’s lungs could have come from the spread of infection from its intestines, the airborne transmission should also be taken into consideration as the initial source of infection, which further illustrates the potential for airborne *Cryptosporidium* transmission in humans.

There are a limited number of respiratory cryptosporidiosis cases reported in the Gulf countries; however, the extent of this type of lung infection has yet to be established in the region. More research is needed to verify the actual risk from cryptosporidial respiratory tract infections in the Gulf human and animal populations. Already researchers have shown that breathing has the potential to release aerosols from infective individuals into a room [181]. Recently, investigators have reported the use of computational multiphase fluid dynamics and heat transfer to demonstrate the transport, dispersion, and evaporation of saliva and respiratory particles that can arise from the human cough. They have calculated the effect of wind speed on social distancing safety measures during the COVID-19 pandemic. Interesting to note that when they considered all the environmental conditions, they concluded that a safety measure of 2 m between people is insufficient to completely prevent the inhalation of respiratory particles and droplets [179].

It is advisable that when managing patients infected with enteric cryptosporidiosis, particularly in those who have unexplained respiratory symptoms, they should be isolated or given face masks as a precautionary measure to avoid the spread of *Cryptosporidium* oocysts from their respiratory droplets that can be released when coughing or sneezing. Therefore, patients should be advised to always protect their mouths and noses with handkerchiefs when they cough or sneeze.

## 5. Conclusions

Routine diagnostic and surveillance systems are an important part of public health and the treatment of infectious diseases. They have the power to prevent outbreaks and save lives. *Cryptosporidium* and other parasites have not yet been included in the routine diagnostic and surveillance systems of the Gulf regions. However, the apparent disease burden of parasitic infections and other infectious disease has been cited in the literature from these GCC countries. The limited number of reports that was found in this review indicate that *Cryptosporidium* has almost infected every element of the Gulf region; in addition, the burden of this parasite in humans, animals, and food and water supplies is starting to show up more in the published literature.

*Cryptosporidium* has definitely had a negative impact on the economic prosperity and public health in this region, while much of this burden has been underrecognized, underestimated, and underreported in reports. Many of the risk factors for contracting *Cryptosporidium* are an everyday reality for the inhabitants of the GCC countries.

The most vulnerable groups (e.g., children under 5 years and immunocompromised individuals) are the most susceptible to the adverse effects of cryptosporidiosis and should be protected from this preventable infectious disease. Molecular analysis of *Cryptosporidium* from isolates in the Gulf population have revealed the presence of zoonotic and anthroponotic transmission according to the published reports.

Desalinated water and other drinking water sources in the GCC countries have been found to be contaminated with *Cryptosporidium* oocysts. Defective waste management systems and water treatment plants have been found to be a source of septic pollutants in the drinking water supplies.

Camels and other animals often accompany owners to sporting events and leisure activities in the GCC countries, which has been noted to be a significant source of zoonotic cryptosporidiosis in the region. *Cryptosporidium* outbreaks have been recorded in animals by incidental or accidental findings. Authors have commented that many of these cryptosporidiosis outbreaks in animals from Gulf region continue be undetected or underreported in the literature.

Expatriates workers have been found to be a source of “imported” *Cryptosporidium* infection via food handling and poor hygiene; however, more detailed investigations are needed to compare this group of the population with the native inhabitants of the area.

Large quantities of food are imported to feed the expanding work force in the Gulf region. Food is usually imported from low socioeconomic countries that are associated with a higher risk of contracting cryptosporidiosis due to their social and economic situation.

Food safety and security legislation has been enacted in the GCC countries to prevent foodborne outbreaks in the region. However, their regulatory standards for imported food still lack many of the parasites known to cause outbreaks, such as *Cryptosporidium*, in their screening protocols. This needs to urgently change so that the prosperity of the local economy and the most vulnerable populations are protected from the burden of foodborne outbreaks in the Gulf region.

Imports of animals, such as cattle, may impact the known epidemiological importance of the release and transmission of *Cryptosporidium* oocysts. A new animal reservoir with its related implications is generated in the GCC countries due to political tensions in the region.

Further research is required to quantify the influence of transmission parameters such as the infective airborne respiratory droplets of *Cryptosporidium* on disease burden, along with those of other pathogenic microorganisms. More research is needed for the development of highly effective disinfection methods to treat *Cryptosporidium* contamination in swimming pools and the water supplies, e.g., bottled water and ground water.

The GCC countries should include *Cryptosporidium* and other parasitic pathogens in their public health protocols for the routine screening of infectious diseases in human and animal faecal samples who have contact with the food and water supply in order to avoid outbreaks. The airborne transmission of *Cryptosporidium* oocysts is highlighted due to the particularly windy and dry environmental conditions associated with this region. The wind has the power to circulate minuscule particles of dried infective faecal matter in the surrounding areas that can poses a threat to human and animal health.

More published research is needed on the epidemiology of *Cryptosporidium* in order to determine the true prevalence of this parasitic pathogen in the GCC countries.

## Figures and Tables

**Table 1:** Reports with details of *Cryptosporidium* infection in the Gulf Cooperation Council (GCC) countries and related information.

Country	Serial No. of Reports	City	Type of Residents	Patients Classification	Most Affected Age	Symptoms	Method of Detection	Prevalence No. of Infected/No. of Total (%)		Reference
Bahrain	-		-	-	-		-	-		-
Kuwait	1	Safat	Inhabitants	Children	4–8 Y	Diarrhoea	MZN, PCR-RFLP	87/2548	* (3.4%)	[49]
	2	Farwanyia	Inhabitants	Children	>2 Y	Diarrhoea, nausea, vomiting	MSMB, DFA	51/3549	(10%)	[39]
	3	Jabryia	NM	Hospitalized patients	NM	Diarrhoea	xTAG GPPA	1/109	(0.9%)	[61]
	4	Safat	Inhabitants	Children	2–3 Y	GIT symptoms mainly diarrhoea	MZN, PCR-RFLP	58/62	(94%)	[50]
	5	Farwanyia	NM	ICCT children	<3 Y	Diarrhoea, dehydration, fever, abdominal pain	NM	35/2205	(1.6%)	[62]
Oman	1	Muscat	Expatriates	Food handlers	NM	Asymptomatic	KKT, TS, APFM	2/100	(0.2%)	[63]
	2	Muscat	Inhabitants and expatriates	Children	<2 Y	Diarrhoea, fever, vomiting	MZN, APFM	16/807	(1.9%)	[64]
Qatar	1	Doha	Inhabitants and expatriates	Hospitalized paediatrics	<2.5 Y	Chronic diarrhoea	q-PCR	90/580	(15.5%)	[46]
	2	Doha	Expatriates	Immigrants	23–29 Y	Asymptomatic	q-PCR	38/839	(4.5%)	[45]
SA	1	Dammam and Alkhobar	Inhabitants	Children and adults	2 Y	Diarrhoea	MZN, APFM	2/321	(0.6%)	[65]
	2	Al-Taif	Inhabitants	Children and adults	<5 Y	Diarrhoea	MZN, LFIT, Con-PCR	21/180	(11.6%)	[66]
	3	Gizan and Madinna	NM	Children	<2 Y	Diarrhoea and asymptomatic	MZN, MSMB, EIA, PCR-RFLP, sequencing	103/1641	(6.3%)	[67]
	4	Al-Taif	NM	Children and adults	<5 Y	Acute diarrhoea	xTAG GPPA	14/163	(8.5%)	[68]
	5	Dhahran	NM	Various ages	NM	Abdominal symptoms mainly diarrhoea	MZN, DFA	66/100	(66%)	[69]
	6	Mekkah	Inhabitants	Children < 14 y	<5 Y	Diarrhoea	MZN, ICT, PCR-RFLP	23/1380	(1.7%)	[70]
	7	Jeddah	Inhabitants	Children	<5 Y	Asymptomatic and diarrhoea	MZN	29/253	(11.5%)	[71]
	8	Riyadh	NM	HIV patients	2–10 Y	Diarrhoea and non-diarrhoea	MZN	11/136	(8.1%)	[72]
	9	Makkah	NM	People attending clinics around the Holy Masjid	<30 Y	Enteritis	MZN	5/183	(2.7%)	[60]
	10	Riyadh	NM	Children <10 y	NM	Diarrhoea	MSMB	2/174	(1.1%)	[73]
	11	Hail	NM	School children	NM	Diarrhoea and non-diarrhoea	MZN	74/200	(37%)	[74]
	12	Riyadh	Inhabitants	ICP	16–40 Y	Chronic diarrhoea Malnutrition	MZN, ELISA	285/408	(69.9%)	[75]
	13	Riyadh	Inhabitants with few expatriates	In-and out-patients	0–10 Y	NM	MZN	6/5987	(0.1%)	[76]
	14	Al-Taif	NM	Children	<10 Y	NM	MZN, AP-PCR, Sequencing	11/100	(11%)	[77]
	15	Jeddah	NM	Children	NM	NM	MZN, APFM, ELISA, Nested PCR, PCR-RFLP	35/500	(7%)	[78]
UAE	16	Hail	Inhabitants and expatriates	Saudi and non-Saudi patients	NM	Asymptomatic	MZN	25/130	19.2%	[79]
	1	Sharjah	Expatriates	Adults	≤25 Y	Asymptomatic	q-PCR	26/134	(19.4%)	[48]
	2	Al-Ain	Expatriates	Adults	30–39	Asymptomatic	MZN, Con-PCR	16/86	(18.6%)	[47]
	3	Al-Ain	NM	ICP and ICTT Children	<5	Diarrhoea Fever Vomiting ALA	MZN	7/140	(5%)	[80]

* The prevalence is among children with diarrhoea. NM: Not Mentioned; Y: Years; GPPA: Gastrointestinal Pathogen Panel Assay; KKT: Kato Katz technique; TS: Trichrome stain; DFA: Direct Immunofluorescent Assay; MSMB: Modified Safranine Methylene Blue stain; APFM: Auramine Phenol Fluorescent Method; ELISA: Enzyme Linked Immunosorbent Assay; Con-PCR: Conventional PCR; LFIT: Lateral Flow Immune Test; EIA: Enzyme Immune Assay; ICT: Immunochromatography; HIV: Human Immunodeficiency Virus; ICP: Immune Compromised Patients (organ transplantation, cancer and HIV); ICCT: Immune Competent; AP-PCR: Arbitrarily Primed PCR; PCR-RFLP: Restriction Fragment Length Polymorphism; q-PCR: Real Time PCR; ALA: Acute Lymphoblastic Anaemia.

**Table 2:** Genotyping and subtyping of *Cryptosporidium* in the reports of the GCC countries.

Country/City	Target Population	Methods Used	Gene Target	Reported Genotypes	No. of Cases within the Species (%)	Subtypes Allele Family	No. of Cases within Each Allele	Reference
Kuwait/Safat	Children with diarrhoea	PCR-RFLP	18S rRNA Gp60	*C. parvum*	61/83 (73.5%)	* C. parvum *		[49]
						IIa	29	
				*C. hominis*	22/83 (26.5%)	IId	20	
						IIc	12	
						* C. hominis *		
				Mixed “*C. parvum* and *C. hominis*”	4/83 (4.8%)	Id	12	
						Ia	8	
						Ie	2	
						Mixed infection	NI	
Kuwait/Safat	Symptomatic children	PCR-RFLP Sequencing	18S rRNA Gp60	*C. parvum*	58/62 (93.5%)	*C. parvum*		[50]
						IId	29	
						IIa	28	
				*C. hominis*	3/62 (4.8%)	IIc	1	
				Mixed “*C. parvum* and *C. hominis*”	1/62 (1.6%)	IIf	1	
						*C. hominis*		
						Ib	2	
						Id	1	
						Ie	1	
Qatar/Doha	Hospitalized paediatrics with diarrhoea	qPCR Sequencing	18S rRNA Gp60	*C. parvum*	83/90 (92.2%)	*C. parvum* ^1^		[46]
				*C. hominis*	4/90 (4.4%)	IId	83	
				*C. meleagridis*	1/90 (1.11)	*C. hominis*		
				Mixed “*C. parvum* and *C. hominis*”	1/90 (1.11%)	Ib	4	
				Mixed “*C. parvum* and *C. meleagridis*”	1/90 (1.11%)			
Qatar/Doha	Asymptomatic Immigrants	qPCR Sequencing	18S rRNA Gp60	*C. parvum*	30/38 (80%)	*C. parvum*		[45]
				*C. hominis*	1/38 (2.6%)	IId	30	
				Mixed “*C. parvum* and *C. hominis*”	4/38 (10.5%)	* C. hominis *		
				Mixed “*C. parvum* and *C. meleagridis*”	3/38 (7.9%)	Ie	1	
SA/Makkah	Children with diarrhoea	PCR-RFLP	18S rRNA	*C. hominis*	(81.1%)	NI	-	[70]
				*C. parvum*	(16.7%)	NI	-	
SA/Gizan and Maddina	Children with diarrhoea and asymptomatic children	PCR-RFLP Sequencing	18S rRNA Gp60 HSP70	*C. parvum*	79/101 (78.2%)	* C. parvum *	NM	[67]
						IId		
				*C. hominis*	13/101 (12.9%)	IIa		
						IIc		
				Mixed “*C. parvum* and *C. hominis*”	8/101 (7.92%)	* C. hominis *	NM	
						Ib		
						Ie		
SA/Al-Taif	Children form different hospitals and laboratories	AP-PCR Sequencing	18S rRNA	*C. parvum*	11/100 (11%)	NI	NI	[77]
SA/Jeddah	Asymptomatic Children	Nested PCR and PCR-RFLP	18S rRNA COWP Gp60	*C. parvum*	15/35 (42.9%)	NI	NI	[78]
				*C. hominis*	13/35 (37%)			
				*C. meleagridis*	1/35 (2.9%)			
				*C. muris*	1/35 (2.9%)			

^1^ Further sub-classification led to 10 different subtypes; NI: Not Identified; NM: Not Mentioned; PCR-RFLP: Polymerase-Chain-Reaction-Restriction Fragment Length Polymorphism; AP-PCR: Arbitrarily primed P.

**Table 3:** Detection of *Cryptosporidium* in water resources of the GCC countries.

Country/City	Type of Contaminated Water	Method Used	No. of Contaminated/No. of Total	Genotyping/Subtyping	References
Kuwait/Safat	Overhead water tanks	IFT PCR-RFLP of 18S rRNA	1/5	^2^*C. parvum* subtype IIa	[49]
SA/Al-Taif	Underground water (UW) from wells	Nested PCR	UW 7/96	NP	[52]
	Desalinated water (DW) from tanks in private houses		DW 8/72		
	Bottled water (BW)		BW 0/60		
^1^ SA/Tabuk	Bottled water	Filtration MZN ELISA	6/36	NP	[111]
^1^ SA/Mekka and Jeddah	Tap water	Double centrifugation MZN	Schools 13/44	NP	[112]
	Bottled water		Houses 33/122		
	Ablution water		Mosques 31/79		
UAE/Dubai	School swimming pool	IFT	5/5	NP	[113]
UAE/Dubai	Irrigation water (IW) of public parks	IFT	IW 17/18	NP	[114]
	Chlorinated water (CW) samples from the sewage treatment plant		CW 5/5		

^1^ The results of these studies regarding types of water were marginal. ^2^ Five members of the same family who lived around this camp suffered from infection with *C. parvum* subtype IIa. IFT: Immunofluorescence; NP: Not Performed.

**Table 4:** *Cryptosporidium* infection in animals of the GCC countries.

Country/City	Type of Animal	No. of Infected/No. of Total	Method Used	Genotyping of *Cryptospori-Dium*	Subtyping of *Cryptosporidium*	Reference
Kuwait/different areas	Sheep Goats	38/334 16/222	MZN EIA PCR-RFLP Sequencing	*C. parvum*	IIdA20G1	[95]
				*C. ubiquitum*	IIaA15G2R1	
				*C. xiaoi*	XIIa	
Kuwait/Kabd, Salmi, Abdelli and Wafra areas	Calves Goat kids Lambs	15/40 12/57 16/128	MZN ICT	NP	NP	[135]
Kuwait/Sulaibiya	Newborn calves	31/80	MZN	NP	NP	[136]
^1^ Oman/Muscat	Goats	238/238	IFT MZN H&E histopathology TEM SEM	*C. parvum*	NP	[137]
SA/Riyadh	Goats	24/72	MZN ELISA	NP	NP	[100]
	Sheep	15/58				
	Camels	20/49				
^2^ SA/Riyadh	Camels	5/33	NM	NP	NP	[138]
^2^ SA/Riyadh	Arabian Oryx	NM	NM	NP	NP	[139]
UAE/Dubai	Falcons	2/2	MZN PCR Sequencing	*C. parvum*	NP	[140]
^1^ UAE/Dubai	Stone curlew	19/29	MZN ICT H&E histopathology PCR-RFLP	*C. parvum* in two samples tested	NP	[141]

^1^ The studies were performed on post-mortem animals due to cryptosporidiosis outbreak. ^2^ The studies were only available in the form of abstracts with no information on methodology and/or prevalence. NP: Not Performed; NM: Not Mentioned; MZN: Modified Ziehl Nelseen; ICT: Immunochromatography; H&E: Haematoxylin and Eosin stain; TEM: Transmission Electron Microscopy; SEM: Scanning Electron Microscopy; PCR-RFLP: Restriction Fragment Length Polymorphism PCR; EIA: Enzymatic Immunoassay; IFT: Immune Fluorescent Technique; ELISA: Enzyme Linked Immune Assay.
